# Impact of a board-game approach on current smokers: a randomized controlled trial

**DOI:** 10.1186/1747-597X-8-3

**Published:** 2013-01-17

**Authors:** Yasser Khazaal, Anne Chatton, Roberto Prezzemolo, Fadi Zebouni, Yves Edel, Johan Jacquet, Ornella Ruggeri, Emilie Burnens, Grégoire Monney, Anne-Sylvie Protti, Jean-François Etter, Riaz Khan, Jacques Cornuz, Daniele Zullino

**Affiliations:** 1 Division of Addictology, University Hospitals of Geneva, Rue Verte 2, Geneva 1205, Switzerland; 2 ECIMUD, Hôpital Pitié Salepêtrière, Paris, France; 3 Institute of Social and Preventive Medicine, University of Geneva, Geneva, Switzerland; 4 Department of Ambulatory Care and Community Medicine, University Hospital, Lausanne, Switzerland

**Keywords:** Smoking, Smoking cessation, Cognitive behavior therapy, Motivational interviewing, Board games

## Abstract

**Background:**

The main objective of our study was to assess the impact of a board game on smoking status and smoking-related variables in current smokers. To accomplish this objective, we conducted a randomized controlled trial comparing the game group with a psychoeducation group and a waiting-list control group.

**Methods:**

The following measures were performed at participant inclusion, as well as after a 2-week and a 3-month follow-up period: “Attitudes Towards Smoking Scale” (ATS-18), “Smoking Self-Efficacy Questionnaire” (SEQ-12), “Attitudes Towards Nicotine Replacement Therapy” scale (ANRT-12), number of cigarettes smoked per day, stages of change, quit attempts, and smoking status. Furthermore, participants were assessed for concurrent psychiatric disorders and for the severity of nicotine dependence with the Fagerström Test for Nicotine Dependence (FTND).

**Results:**

A time × group effect was observed for subscales of the ANRT-12, ATS-18 and SEQ-12, as well as for the number of cigarettes smoked per day. At three months follow-up, compared to the participants allocated to the waiting list group, those on Pick-Klop group were less likely to remain smoker.

Outcomes at 3 months were not predicted by gender, age, FTND, stage of change, or psychiatric disorders at inclusion.

**Conclusions:**

The board game seems to be a good option for smokers. The game led to improvements in variables known to predict quitting in smokers. Furthermore, it increased smoking-cessation rates at 3-months follow-up. The game is also an interesting alternative for smokers in the precontemplation stage.

## Introduction

Although most smokers are informed about the disease and death risks associated with smoking, this awareness is not always sufficient to induce behavioral change [1]. Furthermore, despite the efficacy of nicotine replacement therapy (NRT; [2], bupropion [3], varenicline, and behavioral approaches [4], these treatments are used by only a minority of smokers [5,6], and the number of sustained smoking cessation attempts remains insufficient. This is probably at least partly linked to attitudes towards smoking and smoking cessation [7]; to smokers’ lack of knowledge about, or negative attitudes towards, treatments [8]; and to lack of self-efficacy (confidence in one’s own ability to refrain from smoking in a relapse situation); [9]. For example, a positive attitude towards NRT and an increase in self-efficacy may lower the consumption of cigarettes and increase quitting in smokers [8,10,11]. More specifically, positive attitudes towards NRT are associated with more frequent use of NRT [8]. Thus, the development of new instruments able to have an impact on these dimensions, and which are acceptable by a wide range of smokers, including those who do not want to quit, is of particular interest.

A board game may be a good approach for modifying attitudes and cognitions. Board games probably excite curiosity, as well as emotional and intellectual investment in a secure place (“the game”). They do so in a decentered way that does not induce feelings of guilt, because participants do not have to talk about their individual problems. Board games may also facilitate communication between players. Furthermore, games can be effortlessly disseminated. Games have already been used in another health-related domain with promising results [12].

The board game “Pick-Klop” has been created with this in mind. Its objectives are to (1) inform smokers about smoking and smoking cessation in a way that does not make them feel guilty; (2) increase smokers’ confidence in their ability to stop smoking (self-efficacy); (3) modify attitudes (i.e., perceived advantages and drawbacks) towards smoking and towards tobacco dependence treatments; and (4) help or lead smokers during smoking reduction and smoking abstinence processes.

A first assessment of this game was carried out in 51 patients hospitalized in a psychiatric clinic [13] and concluded to the acceptability of the game and to a favorable impact on intentions to stop smoking. This first study and a second non-controlled study (in press) in a general population of smokers led to improvements in the design and characteristics of the game after a qualitative analysis of the smokers’ comments.

The current study aimed to assess, with a randomized controlled design, the impact of two sessions of this game on a sample of current smokers who did or who did not ask for smoking cessation (precontemplation, contemplation, or preparation stages).

## Methods

### Study design

We conducted a prospective open-label randomized controlled trial with follow-up after 3 months. Eligible smokers were randomly allocated to one of three treatments: two sessions of 1.5 hours each of the Pick-Klop game, two sessions of 1.5 hours each of psychoeducation, or a waiting list. Pick-Klop and psychoeducation sessions were given in a group format once a week for 2 weeks. The randomization ratio was 2:1:1 to obtain more participants in the Pick-Klop experimental condition. A permuted block randomization procedure was done by a research assistant. There was no blinding assessment. The measures were, however, based on self-report questionnaires.

Information related to the study was given in written and oral form by the psychologists working as research assistants. After inclusion criteria were checked, participants gave their written informed consent to the research assistants. The study was approved by ethics commissions in Lausanne, Geneva, and Valais, Switzerland.

### Participants

To be included in the study, participants had to be adults (18–65 years) who were current daily smokers (smoking daily during the last month) and had to give written informed consent. Exclusion criteria were limited to mental retardation and several acute psychiatric disorders that may compromise participation in the Pick-Klop group (acute psychotic episode, manic episode, and moderate or severe depressive episode).

Smokers were recruited between 2007 and 2010 by advertising in local newspapers, and among employees and students at schools and universities.

After preliminary e-mail or phone contact, potential participants received information about the study. A total of 248 current smokers (60% of those who sent an e-mail or asked by phone for further information in response to the advertising) were then assessed for study inclusion. Only two of them were not included because of exclusion criteria and six refused study participation. As a result, 240 smokers were eligible and participated in the study, 65% of whom were women.

**Figure 1:**
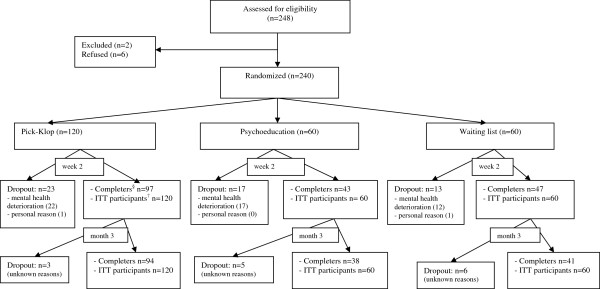
Flow chart of participants through each stage of the experiment.

All participants who fulfilled the inclusion criteria and signed the consent form were randomized as follows: 120 participants in the Pick-Klop group, 60 in the psychoeducation group, and 60 in the waiting-list group (Figure 1).

### Sample size estimation and randomization procedures

This study derives from a more complex study that was designed for six groups of participants (three groups of adults aged between 18 and 65: Pick-Klop, psychoeducation, and waiting list; and three groups of teenagers under 18: Pick-Klop, psychoeducation, and waiting list) and four repeated measures (one baseline and three follow-up measures). From the outcome of the psychoactive benefits of smoking, a domain of Attitudes Towards Smoking Scale (ATS-18), it was hypothesized that a clinically meaningful difference of 0.7 between the Pick-Klop and the psychoeducation group means after treatment could be detected with 80% power. Hence, after inputting the other values in the formula by Diggle, Liang, and Zeger (Analysis of Longitudinal Data, 1994), namely, type I error rate: α = 0.05, measurement variation: σ^2^ = 9.0, number of repeated observations per person: n = 4, and correlation among the repeated observations: *p* = 0.3, the estimated sample size for this design was about 100 persons per group. In order to obtain a balanced design for adult participants, the number of persons in the Pick-Klop group was brought to twice as many as the number of persons in the two control groups put together. Hence, given this 2:1:1 ratio, the final sample size for the Pick-Klop group was 200 participants. Because of recruitment difficulties, however, the randomization procedures generated by http://www.randomization.com stopped prematurely at 30 permuted blocks of 8, thus leaving a sample of 240 adult participants for the analysis. The number of repeated measures also had to be revised downward to three.

### Interventions

#### The Pick-Klop board game

The Pick-Klop game (an informal expression for “pick a cigarette”) includes more than 300 cards with questions, each with three response options. The questions cover the following: (1) smoking and tobacco history, (2) tobacco components and their biological effects, (3) reinforcement mechanisms involved in smoking addiction, (4) cognitive and behavioral mechanisms involved in the maintenance of smoking, (5) smoking cigarettes as a coping strategy, (6) costs of tobacco addiction and the benefits of quitting smoking, (7) stages of change, (8) cognitive and behavioral mechanisms involved in behavioral change, and (9) medications and treatments that help during smoking cessation.

**Figure 2:**
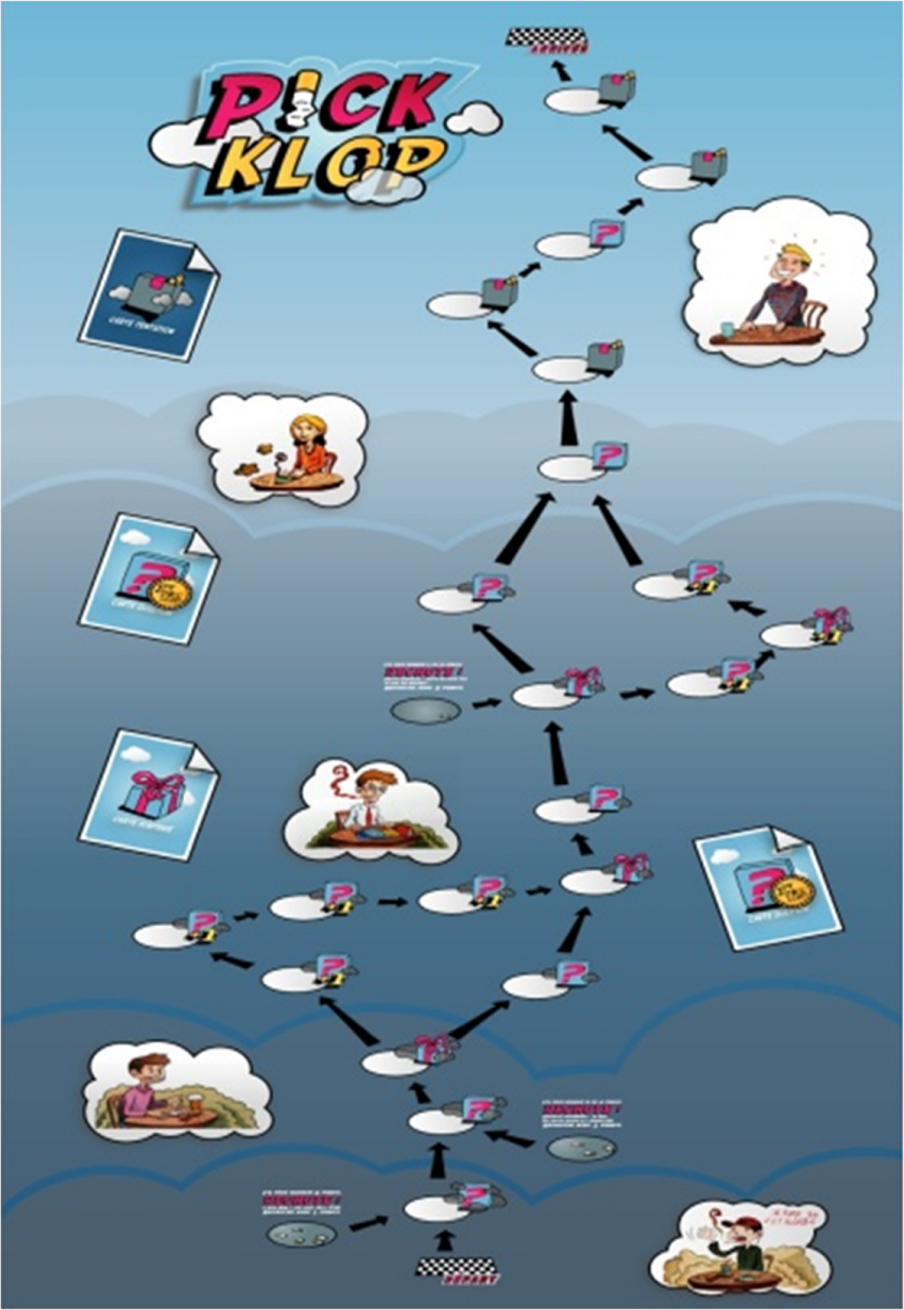
The Pick-Klop game board.

Participants play in groups of two to six. The game board (Figure 2) introduces different characters in different stages of change. Players move their pawns by throwing dice. According to the score obtained, players draw a card in one of the following categories: question, surprise, or temptation. If they answer the question cards correctly, players may gain points. Surprise cards add amusement, allowing players to obtain a gift or secret cards that allow them to help or block another player during play at the moment of their choice. The number of temptation cards increases at the end of the game board. These cards illustrate lapse and relapse processes, as well as relapse prevention strategies. Different lapse/relapse situations are presented, each with two ways of facing the situation: smoking or adopting another behavior. To pass a temptation box, players have to pay points; if they do not have enough points (a metaphoric illustration of inadequate preparation for how to behave in relapse situations), players move to one of three different levels of relapse boxes. In each of these boxes, participants receive bonus points and pursue the game and may win a play. The winner is the first participant to reach the last game box. Participants may choose between shorter or longer ways to attain the end point. Shortcuts may help a player to win quickly, but they involve a possibly higher risk of relapse (fewer questions and then possibly fewer points to pass the temptation boxes). A full game play usually takes between 15 and 45 minutes.

During each game session, the cards are selected randomly. Therefore, in each group, the cards used vary widely. The high number of cards in each category allows participants to explore the main aspects of smoking and smoking-cessation processes during each session and to consider a variety of situations and answers when playing repetitively.

#### Psychoeducation group

Trained psychologists gave two standardized group sessions (1.5 hours each) of psychoeducation (with a slide show) about smoking and smoking cessation. They presented information on smoking, smoking costs (health risks), dependence criteria, motivational stages of change, and available treatments for smoking cessation and discussed these topics with participants.

#### Waiting-list group

Participants on the waiting list, as well those allocated to the other groups, received a Pick-Klop game board after the third month’s assessment.

### Measurements

At inclusion, participants were assessed for socio-demographic characteristics and psychiatric disorders by using the Mini International Neuropsychiatric Interview (MINI; [14]. Psychiatric disorders were assessed to establish exclusion criteria and also in consideration of the frequency of psychiatric disorders among smokers. Population-based studies have repeatedly found that daily smoking is more common among individuals with psychiatric disorders, including mood disorders, than among individuals with no psychiatric diagnosis [15-17]. Furthermore, smokers with psychiatric disorders appear to be less likely to quit smoking than those without such disorders [18]. In consideration of the inclusion and exclusion criteria, we expected the present study on current smokers from the community to include a substantial proportion of persons with psychiatric disorders.

Participants were also assessed for past smoking cessation attempts, smoking environment (living with smokers), nicotine dependence using the Fagerström Test for Nicotine Dependence (FTND; [19,20], and other smoking-related attitudes and behaviors. Several measures related to smoking behavior were repeated at follow-up after 2 weeks (after treatment sessions) and after 3 months. These measures included the following: stage of change [21], (ATS-18) [1], Smoking Self Efficacy Questionnaire (SEQ-12); [22], Attitudes Towards Nicotine Replacement Therapy scale (ANRT-12); [23], smoking status, occurrence of new smoking cessation attempts, methods used to stop smoking, and number of cigarettes smoked per day.

At the end of the second Pick-Klop session, participants also answered questions on satisfaction with the game, using an adaptation of Attkisson’s Client Satisfaction Questionnaire (8-item version, CSQ-8); [24].

The stage of change was assessed with a “yes” or “no” question related to the participant’s intention to quit smoking (i.e., precontemplation: no intention to quit smoking in the next 6 months; contemplation: seriously considering quitting smoking in the next 6 months; preparation: have decided to quit smoking in the next 30 days; and action: stopped smoking).

Smoking status was assessed by self-report. Included participants were daily smokers (smoking daily during the last month) and were considered non-smokers after at least 1 month without smoking for the assessment made at month 3 (T2). Participants were considered non-smokers at T1 (Week 2) if they stopped smoking at least one day before the assessment (initiation of smoking-cessation).

SEQ-12 is a two-dimensional scale that measures confidence in aptitude to refrain from smoking when facing external stimuli (six items: e.g., being with smokers) and internal stimuli (six items: e.g., feeling anxious; [22]. Smokers were asked to indicate whether they were sure that they would refrain from smoking in each situation. Response options were coded on a Likert scale from not at all sure (score: 1 point) to absolutely sure (score: 5 points).

The three subscales of the ATS-18 measure perceptions of adverse effects of smoking (10 items), psychoactive benefits of smoking (four items), and pleasure of smoking (four items). Answers were given on a 5-point Likert scale from totally disagree (score: 1) to fully agree (score: 5).

ANRT-12 is composed of two subscales that measure the perception of the advantages (eight items) and drawbacks (four items) of NRT, respectively [23]. Participants answered the questions on a 5-point Likert scale ranging from strongly disagree (score: 1) to fully agree (score: 5). A sixth response option is “I don’t know.” A further subscale of the ANRT-12 is related to the sum of “I don’t know” answers. The total score for each subscale is obtained by adding the scores obtained for each item. A higher score on a subscale means higher involvement in the subscale.

ATS-18, SEQ-12, and ANRT-12 have good psychometric properties [1,22,23]: test-retest correlation coefficients range between 0.75 and 0.95, and internal consistency coefficients (Cronbach’s alpha) range between 0.75 and 0.95 for all scales.

### Primary and secondary outcomes

The primary outcomes were the following ones:

i. ANRT-12 score assessed at baseline, and then 2 weeks and 3 months later

ii. ATS-18 score assessed at baseline, and then 2 weeks and 3 months later

iii. SEQ-12 score assessed at baseline, and then 2 weeks and 3 months later

The secondary outcomes were the following ones:

iv. Smoking status assessed at week 2 and month 3

v. Number of cigarettes smoked per day assessed at baseline, and then 2 weeks and 3 months later

vi. Number of quit attempts assessed at baseline, and then 2 weeks and 3 months later

vii. Stage of change assessed at baseline, and then 2 weeks and 3 months later

viii. Satisfaction of the Pick-Klop user assessed at week 2.

In the text, baseline, 2-week, and 3-month measures will be referred to henceforth as T0, T1, and T2 measures, respectively.

### Statistical analysis

Preexisting differences between the three study groups in participants’ characteristics at baseline, as well as potential attribution bias between completers and non-completers, were checked.

In this regard, for quantitative measures, one-way analysis of variance (ANOVA) or Student t-tests were used as appropriate, whereas for categorical measures, we used the Pearson’s chi-square or Fisher’s exact tests. The impact of the game was studied through changes over time occurring in the outcome measures between the three study groups as follows.

For quantitative outcomes (ANRT-12, ATS-18, SEQ-12, number of cigarettes smoked, number of quit attempts), we used repeated measures ANCOVAs (analysis of covariance), with treatment group (Pick-Klop vs. psychoeducation vs. waiting list) as the factor of interest while controlling for age and gender which were found differently distributed across groups at baseline. Preliminary checks were conducted to ensure that the assumptions of normality, linearity, homogeneity of variances and homogeneity of regression slopes were satisfied. When either of these conditions was not met, appropriate transformations such as categorization of the covariate were done, notwithstanding some loss of information, and repeated measures ANOVAs conducted instead.

To assess the evolution of categorical outcomes (*stage of change* observed at T0, T1, and T2 and *smoking status* observed at T1 and T2), we used the generalized estimations equation (GEE) approach to handle these correlated data. This procedure fits a model for the dependent variable *stage of change* to the respective *smoking status*, using *treatment group* and *time* as factors. To allow for adequate group size, we dichotomized *stages of change* as follows: participants in the precontemplation stage were grouped in stage 1, while participants in the contemplation, preparation, and action stages were grouped in stage 2. Because the model specification assumes a binomial distribution of the outcomes, we chose a logit link function, which relates the probability of these outcomes to a linear combination of the predictors. As a goodness-of-fit criterion, we used The Quasi-likelihood under Independence Model Criterion (QIC) to select between two models: one with the main effects only and one with the main effects together with an interaction term. The model that obtains the smaller QIC is “better” according to this criterion.

In addition, to assess potential factors associated with smoking cessation (smoker vs. non-smoker) at T2, we used a multiple logistic regression model with age, gender, FTND scores, treatment group, stage of change and psychiatric disorders as independent variables. Likewise, selected end-point outcomes, namely ARNT-12, ATS-18 and SEQ-12 scores at T2 were also regressed on the same variables but in multiple linear regression models due to their quantitative nature.

Analyses were done on an intention-to-treat basis, whereby all randomized subjects were included. Quantitative missing data were imputed by the expectation-maximization algorithm, a statistical simulation technique that estimates the averages, the matrix of variance and covariance, and the matrix of correlations for the quantitative variables by using an iterative procedure. After convergence, the missing data were replaced by their obtained estimation and the completed data were then analyzed by the usual methods.

For categorical outcomes, adopting the worst-case scenario, we assumed that everyone who was missing in the intervention groups or in the control group had the worst outcomes. All the statistical tests were two-tailed and at a 5% significance level. Statistical analysis was performed with SPSS (version 18.0, IBM, Chicago, USA).

## Results

### Baseline characteristics of subjects

The mean age of the participants was 31.5 (SD = 12.1) years, the mean baseline cigarette consumption was 15 cigarettes per day (SD = 7.9), and the mean age at onset of smoking was 16.2 years (SD = 2.7). The Fagerström Test for Nicotine Dependence (FTND) mean was 3.2 (SD = 2.1). Among participants, 28.11% had psychiatric disorders according to MINI, most frequently mood disorders. Furthermore, 22.7% of smokers reported cannabis use at least once a week, 77.5% reported regular alcohol consumption, and 6.2% had alcohol dependence according to MINI. At T0, 35% of smokers were in the precontemplation stage of change (had no intention to quit smoking in the next 6 months).

At T0, participants differed on the following variables: gender, age, age at start of smoking, number of cigarettes smoked per day, and stage of change (Table 1).

**Table 1:** Baseline participants’ characteristics by study group

**Characteristic**	**Pick-Klop group (n**_**1**_ **= 120)**	**Psychoeducation group (n**_**2**_ **= 60)**	**Waiting-list group (n**_**3**_ **= 60)**	***p *value**
**Age in years**	**33.7 ±13.4 (19, 66)**	**28.7 ±10.8 (18, 59)**	**30 ±10 (20, 60)**	**0.03**
**Women,%**	**64.2**	**78.3**	**53.3**	**0.02**
**Education,%**				**n.s.**
- **High school or lower**	**14.3**	**1.7**	**13.3**	
- **Professional school**	**27.7**	**40**	**30**	
**University degree**	**58.0**	**58.3**	**56.7**	
**Smoking history**				
- **Age at start of smoking**	**16.9 ±3 (8, 27)**	**15.2 ±2 (11, 20)**	**16 ±2.2 (12, 23)**	**0.03**
- **Years of cigarette smoking**	**16.9 ±13.4 (1, 49)**	**13.5 ±10.2 (3, 40)**	**14.4 ± 10.6 (2, 44)**	**n.s.**
- **No. of cigarettes smoked/day**	**15.8 ±8.3 (3, 50)**	**15.5 ±7.3 (4, 40)**	**12.8 ±7 (1, 32)**	**0.04**
- **No. of previous attempts to quit**	**2.8 ±2.4 (0, 17)**	**3.2 ±2.4 (0, 12**	**2.4 ±2.4 (0, 15)**	**n.s.**
- **Have previously sought help to quit,%**	**35**	**31.7**	**30**	**n.s.**
**Living with a smoker,%**	**47.5**	**56.7**	**58.3**	**n.s.**
**Stage of change,%**				**0.01**
- **Precontemplation**	**38.3**	**20**	**43.3**	
- **Contemplation**	**48.3**	**61.7**	**46.7**	
- **Preparation**	**13.3**	**18.3**	**10.0**	
**Fagerström Test for Nicotine Dependence**	**3.3 ±1.9 (0, 8)**	**3.4 ±2.1 (1, 8)**	**2.8 ±2.4 (0, 10)**	**n.s.**
**Attitudes Towards Nicotine Replacement Therapy Scale**				
- **Perceived advantage**	**19.3 ±8.6 (0, 35)**	**18.8 ±8.8 (2, 32)**	**19.7 ±8.8 (0, 35)**	**n.s.**
- **Drawback**	**9.9 ±4.3 (2, 20)**	**11.3 ±4.4 (4, 20)**	**10 ±4.7 (0, 20)**	**n.s.**
- **“Don’t know” answers**	**2.2 ±2.7 (0, 11)**	**2.4 ±2.9 (0, 8)**	**2.5 ±3.2 (0, 12)**	**n.s.**
**Attitudes Towards Smoking Scale**				
- **Adverse effects of smoking**	**38.6 ±6.8 (15, 50)**	**38.8 ±8.5 (15, 50)**	**39.8 ±5.3 (28, 50)**	**n.s.**
- **Psychoactive benefits of smoking**	**13.9 ±4.1 (4, 20)**	**14.3 ±4.1 (6, 20)**	**14 ±4.3 (4, 22)**	**n.s.**
- **Pleasure of smoking**	**14.4 ±3.8 (4, 20)**	**13.6 ±3.1 (5, 20)**	**14.7 ±3.6 (8, 20)**	**n.s.**
**Smoking Self-Efficacy Questionnaire**				
- **Internal score**	**15.6 ±5.9 (5, 30)**	**14 ±6.0 (6, 30)**	**15.9 ±6.7 (6, 30)**	**n.s.**
- **External score**	**13.8 ±6.3 (4, 30)**	**11.9 ±5.3 (4, 28)**	**13.7 ±7.0 (6, 30)**	**n.s.**
- **Total score**	**29.3 ± 10.8 (10, 60)**	**25.8 ± 9.5 (12, 55)**	**29.8 ± 12.5 (12, 54)**	**n.s.**
**Psychiatric disorder history,%**				**n.s.**
- **Depression/mood disorder**	**19.2**	**10**	**23.3**	
- **Other disorder**	**8.3**	**10**	**10**	

Interval variables are reported by their means and standard deviations, M ± SD, and by their ranges (min, max). Categorical variables are reported by their percentages,%. n.s. = non-significant.

### Dropouts

At T1, 53 participants (22.1%) discontinued the study. Their baseline characteristics were compared with those who continued the study (Table 2). There was a statistically significant difference between completers and non-completers regarding the stage of change (*p* = 0.006), the level of education (*p* = 0.01), and the psychoactive benefits of smoking (*p* = 0.008). By using logistic regression, we observed that, compared with those who continued, participants who dropped out of the study were more likely to be in the preparation stage of change than in the precontemplation stage, had a lower level of education and scored higher on the perception of the psychoactive benefits of smoking. There was no attribution bias since no statistical difference in the distribution of completers and non-completers in the three study groups was observed. At T2, 14 participants did not show up for unknown reasons. In the end, the rate (number) of non-completers was 21.7% (26), 36.7% (22), and 31.7% (19) in the Pick-Klop, psychoeducation, and waiting-list groups, respectively. The details of their distribution are shown in Figure 1.

**Table 2:** Participants’ characteristics by study completion status

**Characteristic**	**Completer (n**_**1**_ **= 187)**	**Non-completer (n**_**2**_ **= 53)**	***p *value**
**Study group,%**			**n.s.**
- **Pick-Klop**	**80.8**	**19.2**	
- **Psychoeducation**	**71.7**	**28.3**	
- **Waiting list**	**78.3**	**21.7**	
**Age in years**	**31.2 ±12.0 (19, 66)**	**32.8 ±12.7 (18, 59)**	**n.s.**
**Gender,%**			**n.s.**
- **Female**	**79.5**	**20.5**	
- **Male**	**75.0**	**25.0**	
**Education,%**			**0.01**
- **Professional school**	**74.7**	**25.3**	
- **University degree**	**83.3**	**16.7**	
**Smoking history**			
- **Age at start of smoking**	**16.2 ±2.6 (8, 27)**	**16.4 ±2.9 (11, 20)**	**n.s.**
- **Years of cigarette smoking**	**15.0 ±12.0 (1, 49)**	**16.4 ±12.0 (3, 40)**	**n.s.**
- **No. of cigarettes smoked/day**	**15.4 ±7.2 (3, 50)**	**14.0 ±10.0 (4, 40)**	**n.s.**
- **No. of previous attempts to quit**	**2.5 ±2.3 (0, 17)**	**3.1 ±2.8 (0, 12)**	**n.s.**
**Have previously sought help to quit,%**			**n.s.**
- **Yes**	**82.9**	**17.1**	
- **No**	**76.9**	**23.1**	
**Living with a smoker,%**			**n.s.**
- **Yes**	**77.0**	**23.0**	
- **No**	**78.8**	**21.2**	
**Stage of change,%**			**0.006**
- **Precontemplation**	**89.3**	**10.7**	
- **Contemplation**	**73.2**	**26.8**	
- **Preparation**	**66.7**	**33.3**	
**Fagerström Test for Nicotine Dependence**	**3.2 ±2.1 (0, 8)**	**3.1 ± 2.1 (1, 8)**	**n.s.**
**Attitudes Towards Nicotine Replacement Therapy Scale**			
- **Perceived advantage**	**19.2 ±8.3 (0, 35)**	**19.4 ±9.8 (2, 32)**	**n.s.**
- **Drawback**	**10.3 ±4.5 (2, 20)**	**10.3 ±4.3 (4, 20)**	**n.s.**
- **“Don’t know” answers**	**2.2 ±2.8 (0, 11)**	**2.7 ±3.2 (0, 8)**	**n.s.**
**Attitudes Towards Smoking Scale**			
- **Adverse effects of smoking**	**38.9 ±6.7 (15, 50)**	**38.7 ±7.8 (15, 50)**	**n.s.**
- **Psychoactive benefits of smoking**	**13.7 ±3.9 (4, 20)**	**15.3 ±4.5 (6, 20)**	**0.008**
- **Pleasure of smoking**	**14.5 ±3.6 (4, 20)**	**13.5 ±3.4 (5, 20)**	**n.s.**
**Smoking Self-Efficacy Questionnaire**			
- **Internal score**	**15.2 ± 6.2 (5, 30)**	**15.8 ±5.9 (6, 30)**	**n.s.**
- **External score**	**13.0 ±6.1(4, 30)**	**14.1 ±6.7 (4, 28)**	**n.s.**
- **Total score**	**28.2 ± 10.9 (10, 60)**	**29.9 ± 11.3 (12, 55)**	**n.s.**
**Psychiatric disorder history,%**			**n.s.**
- **Depression/mood disorder**	**69.8**	**30.2**	
- **Other disorder**	**72.7**	**27.3**	

Interval variables are reported by their means and standard deviations, M ± SD, and by their ranges (min, max).

Categorical variables are reported by their percentages,%. n.s. = non-significant.

### Attitudes Towards Nicotine Replacement Therapy Scale (ANRT-12)

We conducted a repeated ANCOVA analysis for the perceived advantages of NRT. The multivariate results showed a statistically significant overall time effect (*F*_*(2, 234)*_ = 4.3, *p* =0.01). We noted a sharp increase of scores between T0 and T1 (*F*_*(1,235)*_ = 7.0, *p* = 0.009). There also was a treatment group × time interaction (*F*_*(4,470)*_ = 8.3, *p* < 0.0005). Both intervention groups compared to the control group significantly increased their mean between T0 and T1 (*F*_*(2,235)*_ = 16.7, *p* < 0.0005). This mean difference was still significant between T1 and T2 (*F*_*(2,235)*_ = 3.3, *p* = 0.04). Finally, a main group effect was observed (*F*_*(2,235)*_ = 6.0, *p* = 0.003). This translates higher overall means on the dependent variable for Pick-Klop and psychoeducation groups compared to the waiting list group. The covariate age was not linearly related to the dependent variable and therefore provided no statistically significant adjustment. No significant main effect of gender was found.

For the nicotine perceived drawback, the covariate age had to be transformed to a categorical variable due to the heterogeneity of the regression slopes. Based on the histogram, three levels of this variable were identified: 18–24, 25–30 and more than 30 years old. Hence, we conducted a repeated ANOVA. The multivariate results showed a statistically significant time x treatment group effect (*F*_*(4, 460)*_ = 4.4, *p* =0.002). Between T0 and T1 the mean scores for Pick-Klop and waiting list groups increased (*F*_*(2, 230)*_ = 7.9, *p* < 0.0005) while they decreased for psychoeducation. A statistically significant treatment group x age interaction was found (*F*_*(4, 230)*_ = 6.3, *p* < 0.0005). The youngest in the Pick-Klop and waiting list groups had higher mean scores than their counterparts in psychoeducation. No main effect was observed for gender.

Regarding the “don’t know” answer scores, a repeated ANCOVA analysis yielded significant overall time effect (*F*_*(2,234)*_ = 9.0, *p* < 0.0005). The scores decreased between T0 and T1 (*F*_*(1, 235)*_ =18.1, *p* < 0.0005) and between T1 and T2 respectively (*F*_*(1, 235)*_ =5.6, *p* =0.02 ). There also was a treatment group × time interaction (*F*_*(4,470)*_ = 3.2, *p* = 0.01). Between T0 and T1, psychoeducation groups decreased their scores more than Pick-Klop and the waiting list group (*F*_*(2,235)*_ = 5.2, *p* = 0.006). But between T1 and T2, Pick-Klop decreased its scores more than psychoeducation and waiting list groups (*F*_*(2,235)*_ = 4.7, *p* = 0.01). The covariate age was significantly associated with the dependent variable. Moreover a main effect was observed for gender, with higher scores for women than men.

### Attitudes Towards Smoking Scale (ATS-18)

For scores related to the perception of the pleasure of smoking, following an ANCOVA analysis, a multivariate results showed an overall time effect (*F*_*(2, 234)*_ = 8.0, *p* < 0.0005). A decrease of mean scores was observed between T0 and T1 and between T1 and T2 (*F*_*(1, 235)*_ = 7.5, *p* = 0.007 and *F*_*(1, 235)*_ = 4.4, *p* = 0.04 respectively). A time x group interaction (*F*_*(4, 470)*_ = 4.3, *p* = 0.002) was also observed. Between T0 and T1, the mean scores for the two treatment groups decreased while they increased for the waiting list (*F*_*(2, 235)*_ = 5.7, *p* = 0.004). The covariate age was significantly associated with the dependent variable. There was no main effect for gender.

The multivariate results only showed an overall time effect for scores related to the perception of psychoactive benefits of smoking (*F*_*(2, 234)*_ = 6.6, *p* = 0.002). This decrease of scores was mainly observed between T0 and T1 (*F*_*(1, 235)*_ = 11.9, *p* = 0.001). The covariate age was not linearly related to the dependent variable and therefore provided no statistically significant adjustment. On the contrary, there was a significant main effect for gender, with higher scores for women than men.

For adverse effects of smoking, to circumvent the heterogeneity problem of regression slopes, we used categories of age and gender as control variables in a repeated ANOVA. The multivariate results showed an overall time effect (*F*_*(2, 227)*_ = 9.5, *p* < 0.0005). An increase of scores was observed, mainly between T0 and T1 (*F*_*(1, 228)*_ = 14.5, p < 0.0005). A time x treatment group interaction was also observed at the limit of significance (*F*_*(4,456)*_ = 2.4, *p* = 0.05) with an increase for Pick-Klop and waiting list between T0 and T1. An effect of group x age was observed. The eldest in Psychoeducation and waiting list groups had higher mean scores than the youngest in Pick-Klop. Finally, an effect of group and gender showed that women in Pick-Klop and waiting list had higher scores than men. The reverse was true for psychoeducation.

### Smoking Self-Efficacy Questionnaire (SEQ-12)

Self-efficacy towards external stimuli was assessed through an ANOVA for repeated measures using age categories and gender as control variables. An overall time effect was observed (*F*_*(2, 233)*_ = 6.0, *p* = 0.003). It was shown that this effect was accompanied by an increase of scores between T0 and T1 (*F*_*(1, 234)*_ = 4.3, *p* =0.04) and between T1 and T2 (*F*_*(1, 234)*_ = 6.0, *p* = 0.02). There was a main effect for age: older participants had higher mean scores than the youngest. No main effect for gender.

To assess self-efficacy towards internal stimuli, we used an ANCOVA for repeated measures. There was an overall time effect (*F*_*(2, 234)*_ = 6.2, *p* = 0.002). It was shown that this effect occurred mainly between T1 and T2 (*F*_*(1, 235)*_ = 10.4, *p* = 0.001). A treatment group *×* time interaction was observed as well (*F*_*(4, 470)*_ = 3.5, *p* = 0.008). This effect occurred mainly between T1 and T2 (*F*_*(2, 235)*_ = 4.2, *p* = 0.02). The scores were significantly higher for the Pick-Klop group compared with the psychoeducation and the waiting list groups between these times. The covariate age was not linearly related to the dependent variable and therefore provided no statistically significant adjustment. However, a significant main effect was observed for gender, with higher scores for men than women.

### Number of cigarettes smoked per day

A repeated measures ANOVA using age categories and gender as control variables was conducted. The results showed an overall time effect (*F*_*(2 229)*_ = 24.8, *p* < 0.0005). This effect reflects a significant decrease of cigarettes smoked mainly between T1 and T2 (*F*_*(1, 230)*_ = 25.8, *p* < 0.0005). A time x treatment group effect was observed as well (F_(4, 460)_ = 5.2, p < 0.0005). The Pick-Klop group significantly decreased the number of cigarette smoked per day compared to the psychoeducation and the waiting list groups mainly between T1 and T2 (F_(2, 230)_ = 3.5, p = 0.03). Younger participants in psychoeducation smoked more cigarettes per day than their counterparts in Pick-Klop. There was no main effect of gender.

### Number of quit attempts

A repeated measures ANOVA using age categories and gender as control variables was conducted. The multivariate results showed a significant time effect (*F*_*(2, 233)*_ = 31.0, *p* < 0.0005). The mean number of quit attempts increased between T1 and T2 (*F*_*(1, 234)*_ = 9.4, *p* = 0.002). There was a main effect for age: the youngest had a lower number of quit attempts than the eldest. No other effect was observed.

### Stage of change

At T0, 35% of smokers were in the precontemplation stage (had no intention to quit smoking in the next 6 months), 51.3% in the contemplation stage (seriously considered quitting smoking in the next 6 months), and 13.8% in the preparation stage (had decided to quit in the next month). At T1, a new stage emerged: the action stage (participants who stopped smoking). Details for each study group are reported in Table 3.

**Table 3:** Evolution of primary outcome variables by treatment group

**Treatment group**	**Pick-Klop**	**Psycho-education**	**Waiting list**	**Pick-Klop**	**Psycho-education**	**Waiting list**	**Pick-Klop**	**Psycho-education**	**Waiting list**
**Assessment-time**	**Pretest (T0)**	**Pretest (T0)**	**Pretest (T0)**	**Week 2 (T1)**	**Week 2 (T1)**	**Week 2 (T1)**	**Month 3 (T2)**	**Month 3 (T2)**	**Month 3 (T2)**
**Attitudes Towards Nicotine Replacement Therapy Scale**									
- **Perceived advantage**	**19.3 ±8.6**	**18.8 ±8.8**	**19.7 ±8.8**	**25.2 ±7.9**	**25.1 ± 7.7**	**18.3 ± 8.6**	**24.4 ±8.7**	**25.8 ±7.1**	**20.0 ±8.2**
- **Drawback**	**9.9 ±4.3**	**11.3 ±4.4**	**10.0 ±4.7**	**10.5 ± 4.2**	**9.9 ± 3.8**	**11.2 ± 4.6**	**10.9 ±4.6**	**9.8 ±3.7**	**11.0 ±5.3**
- **“Don’t know” answers**	**2.2 ±2.7**	**2.4 ±2.9**	**2.5 ±3.2**	**1.1 ± 1.9**	**0.9 ± 1.7**	**2.5 ± 2.5**	**0.8 ±1.8**	**0.8 ±1.3**	**2.0 ±1.1**
**Attitudes Towards Smoking Scale**									
- **Adverse effects of smoking**	**38.6 ±6.8**	**38.8 ±8.5**	**39.8 ±5.3**	**40.1 ± 7.6**	**40.9 ± 7.6**	**41.4 ± 5.3**	**39.5 ±7.9**	**41.8 ±7.3**	**39.9 ±6.1**
- **Psychoactive benefits of smoking**	**13.9 ± 4.1**	**14.3 ± 4.1**	**14.0 ± 4.3**	**12.8 ± 4.0**	**14.0 ± 3.6**	**13.6 ± 3.8**	**12.8 ± 4.4**	**12.7 ± 3.9**	**13.5 ± 3.6**
- **Pleasure of smoking**	**14.4 ±3.8**	**13.6 ±3.1**	**14.7 ±3.6**	**13.3 ± 3.6**	**12.6 ± 3.3**	**14.9 ± 3.4**	**12.8 ±3.4**	**12.7 ±4**	**15.4 ±4.2**
**Smoking Self-Efficacy Questionnaire**									
- **Internal score**	**15.6 ±5.9**	**14.0 ±6.0**	**15.9 ±6.7**	**16.2 ± 5.8**	**15.1 ± 5.8**	**15.6 ± 5.6**	**18.8 ±6.9**	**14.9 ±7.9**	**15.4 ±5.2**
- **External score**	**13.8 ±6.3**	**11.9 ±5.3**	**13.7 ±7.0**	**14.6 ± 4.8**	**13.7 ± 5.5**	**13.7 ± 5.8**	**16.4 ±6.9**	**14.1 ±7.6**	**14.1 ±6.7**
- **Total score**	**29.3 ± 10.8**	**25.8 ± 9.5**	**29.8 ± 12.5**	**30.8 ± 9.0**	**28.8 ± 10.0**	**29.3 ± 10.3**	**35.2 ±12.4**	**29.4 ±14.9**	**29.7 ±10.2**
**Cigarettes smoked per day**	**15.8 ±8.3**	**15.5 ±7.3**	**12.8 ±7.0**	**15.0 ± 8.7**	**14.9 ± 7.7**	**13.3 ± 12.5**	**10.1 ±8.8**	**12.7 ±10.0**	**11.9 ±7.9**
**Stage of change,**^**1**^**%**									
- **Precontemplation**	**38.3**	**20.0**	**43.3**	**42.5**	**20.0**	**63.3**	**50.0**	**46.7**	**60.0**
- **Contemplation**	**48.3**	**61.7**	**46.7**	**36.7**	**60.0**	**30.0**	**32.5**	**38.3**	**35.0**
- **Preparation**	**13.3**	**18.3**	**10.0**	**17.5**	**18.3**	**3.3**	**2.5**	**3.3**	**1.7**
- **Action**	**- **	**- **	**- **	**3.3**	**1.7**	**3.3**	**15.0**	**11.7**	**3.3**
**Non-smokers,%**	**- **	**- **	**- **	**3.3**	**1.7**	**3.3**	**15.0**	**11.7**	**3.3**

Interval variables are reported by their means and standard deviations, M ± SD. Categorical variables are reported by their percentages,%.

^1^For the purposes of GEE analysis, participants in the precontemplation stage are grouped in stage 1 and participants in all other stages in stage 2.

The evolution of stage of change measures was assessed through the GEEs by which we fitted a model with time and group main effects only, as the inclusion of a time × group interaction term did not seem to give a better fit. The GEE results showed a time effect: compared with participants at T2, participants at T0 and T1 were more likely to be in stage 2 than in stage 1 (*p* < 0.005, OR = 2.04, and CI = [1.45; 2.87] and *p* = 0.004, OR = 1.49, and CI = [1.14; 1.95], respectively). There also was a group effect. With a significance of 0.02, the Pick-Klop and psychoeducation groups were more likely to be in stage 2 than in stage 1 compared with the waiting-list group (OR = 1.8 and CI = [1.1; 2.9].

### Smoking status

Again, according to the QIC value, we chose the main effect model with time and group as factors. The GEE results showed both a time effect and a group effect. Compared with participants at T2, those at T1 were more likely to be smokers (*p* = 0.001, OR = 4.27, and CI = [1.87; 9.77]). Compared with the participants in the waiting-list group, those in the Pick-Klop group were less likely to be smokers (*p* = 0.04, OR = 0.32, and CI = [0.11; 0.96]). There was no significant difference between the psychoeducation and the waiting-list groups as the confidence interval includes the “1” value.

### Prediction of smoking cessation and other cessation-related variables

In order to find possible independent variables that predict smoking cessation at T2, a logistic regression was carried out with the following variables: age, gender, FTND scores, psychiatric disorders (at least one psychiatric disorder or dependence on a substance other than nicotine), treatment group and stage of change at baseline. The results showed that 11% of the variance of the dependent variable was explained by this model. Only treatment group was found to predict smoking cessation at T2. Compared to the waiting list group, Pick-Klop group was less likely to be smoker at T2 (*p* = 0.03, OR = 0.18, and CI = [0.04; 0.86]).

Similarly, predictions of other smoking-related attitudes (ANRT-12, ATS-18, and SEQ-12 subscale scores at T2) by linear multiple regressions with age, gender, FTND scores, stage of change, treatment group and psychiatric disorders as independent variables were significant for treatment group only. For these regressions, the variance ranged from 1% to 14%. Introducing the subscale scores under analysis at T0 as supplementary predictors changed the variance from 7% to 44%. Hence, the scores at T0 were significant predictors of scores at T2.

### Satisfaction of Pick-Klop users

During the sessions, participants laughed frequently in a pleasurable ambiance. They often added personal comments in relation to the cards. The game received a good score on the CSQ-8 by the participants, with a mean score of 23.2 (SD = 3.2) of 32. Only 13.8% of the participants gave a score below 20.

## Discussion

This study showed that Pick-Klop, a board game for smokers, is an acceptable, feasible, and potentially helpful intervention for smokers who wish to quit smoking or for those who do not. At inclusion, 35% of smokers were in the precontemplation stage (had no intention to quit smoking in the next 6 months). The game therefore seems also to be acceptable for smokers in the precontemplation stage.

Furthermore, the study assessed participants for psychiatric and substance abuse disorders, which are common comorbidities among smokers [15-17], showing the acceptability of the game and the study process among participants with comorbid psychiatric or substance use disorders.

The dropout rate was relatively low (22.1%), indicating a good acceptability of the study process and the study treatment procedures. Smokers who dropped out of the study were more likely to be in the preparation stage than in the precontemplation stage. One possible hypothesis is that for people who are in precontemplation, the tools and the game are acceptable, whereas a part of the smokers in preparation may ask for more intensive treatment tools. There is higher dropout among people who scored high on the psychoactive benefits of smoking, probably linked to more difficulty in engaging in a smoking-cessation process. Furthermore, people with a lower level of education dropped out more frequently, possibly due to difficulties regarding several aspects of the game questions or of psychoeducation. Overall, it appears, however, that the smokers reported good satisfaction related to the game.

A favorable time effect was observed for most of the variables under scrutiny. A number of important between group differences were observed.

Across time, between T1 and T2, scores on internal self-efficacy increased for smokers allocated to Pick-Klop more than for the participants allocated to psychoeducation or to the waiting list. The game includes an important number of cards linked to smoking facing internal stimuli. This may explain the more important impact on internal self efficacy than on external self-efficacy.

In addition, smokers in the Pick-Klop and the psychoeducation groups increased their knowledge related to NRT (decrease of “I don’t know” answers). Psychoeductaion seems to have a more important effect between T0 and T1 whereas the effect of Pick-klop seems to be more important between T1 and T2. Furthermore the perceived advantages of NRT increased in pick–klop and psychoeducation groups more than it did among the participants allocated to the waiting-list group.

These observed changes were of high interest in consideration of previous studies showing that a positive attitude towards NRT and an increase in self-efficacy may enhance quitting in smokers, reduce the number of smoked cigarettes, and increase the use of NRT during smoking-cessation attempts [8,10,11].

During the study, a decrease of the scores related to the perception of the pleasure of smoking was observed. This decrease was more important for the participants allocated to Pick Klop and psychoeducation than to the waiting list between T0 and T1 and between T1 and T2.

The study did not find any treatment group effect on the perception of psychoactive benefits of smoking. The mean scores related to the perception of the adverse effects of smoking increased however between T0 and T1 for Pick Klop and waiting list group (at a trend level) more than for the other groups.

Lack of clear treatment group effects on these last two measures is possibly due to the sample size. Furthermore, participants have already a relatively good awareness related to the adverse effects of smoking among participants at inclusion. So it is probably more difficult to increase more this aspect. Psychoactive benefits of smoking were discussed during the Pick Klop game sessions as well as during psychoeducation as possible reinforcement mechanisms involved in smoking addiction and were acknowledged by this way. So the interventions may have an impact on the awareness of the links between these effects and the addictive aspects of the behavior rather than on the strict perception of the effects.

The decrease of the scores related to the perception of the pleasure of smoking may have an impact on further smoking cessation attempts by a modification of the perception of the advantages related to smoking.

Because of the relatively small sample size, stages of change were regrouped into stage 1 (precontemplation) and stage 2 (contemplation, preparation and action). It appears that smokers allocated to the Pick-Klop and psychoeducation groups were more likely to progress towards stage 2 than were participants in the waiting-list group.

Positive group effects on the behavioral measures were also found. For instance, a greater decrease in the number of cigarettes smoked per day was observed for the participants of the Pick-Klop group than for the other groups mainly between T1 and T2.

The observed finding is possibly of interest regarding the previously reported link between the reduction of the number of smoked cigarettes and the observation of further smoking cessation [25].

Furthermore, compared with participants at T1, those at T2 were more likely to be non-smokers. This effect is more important among participants in the Pick-Klop group than among participants in the waiting-list group. There was no significant difference between the psychoeducation and the waiting-list groups.

Thus, the participants allocated to the Pick-Klop group or to the psychoeducation group showed rapid improvement (since T1) in the main non-behavioral outcomes (attitudes towards NRT, self-efficacy, attitudes towards smoking). For some of these outcomes, Pick-Klop seems to have some advantages, particularly on internal self-efficacy and to a lesser extent on the perception of the adverse effects of smoking.

The effect of Pick-Klop on several of these non-behavioral measures appears more clearly between T1 and T2, particularly for internal self-efficacy and knowledge related to NRT. One can hypothesize that the game may enhance change by some connections automatically made by the participants when facing, at distance from the game sessions, situations similar to those encountered during the game sessions (i.e. similarities with situations encountered by the game characters…).

The behavioral effects (reduction of the number of cigarettes smoked per day and smoking cessation) appeared later at T2. One could thus hypothesize that the first non-behavioral modification will lead to later behavioral change. For example, it was previously found that an increase in self-efficacy is associated with further smoking cessation [22]. The participants in Pick-Klop showed greater changes towards smoking cessation than the waiting-list group did. The differences on smoking cessation was however not significant between psychoeducation and the waiting list. The small sample size may contribute to this finding. The game may offer some advantages for the enhancement of behavioral change, possibly by some identification with the game characters.

None of the demographic, psychological, or psychiatric variables studied in the present study were strongly associated with further smoking cessation. This finding is possibly due to the sample size, or to the contribution of other factors to the change process, such as environmental support [26]. Treatment group allocation was however found to predict smoking cessation at T2. Compared to the waiting list group, Pick-Klop group was less likely to be smoker at T2.

The Pick-Klop group performed better than the waiting-list group and as well as or better than the psychoeducation group for the main outcomes. Thus, psychoeducation and the Pick-Klop game may have some advantage on non behavioral (i.e. internal self-efficacy) and behavioral outcome (smoking cessation at T2). The game seems to be a good option, possibly eliciting behavioral change in a wide range of smokers, including smokers in precontemplation or smokers with psychiatric or substance use disorders, as previously suggested by preliminary studies [13].

Despite the randomized controlled design of the present study, some limitations have to be considered, including the open-label design, the absence of long-term follow-up, the absence of biochemical variation in smoking abstinence, and the relatively small sample size. In addition, the particular circumstances of the premature closure of the study because of recruitment difficulties led to an underpowered study. Indeed, with the formula given by Diggle et al., the study could achieve only 70% of power. As power remains a useful statistical measure that acts as a magnifying glass in the detection of an effect size, the research may have sometimes failed to point to a true between-groups difference due to lack of power. The modification of the study objectives (in terms of the number of included participants) was discussed with an independent referral extern to the study group (study founding contributor) and was considered as an acceptable option.

The difficulties related to the recruitment process were probably due to the characteristics of the study which was may be too much demanding in time for smokers who were not asking for help. The game in itself seems to be appreciated by the participants. One of the possible advantages of a game such as Pick-Klop is to offer smokers an alternative way to learn about information and treatment. Further studies may specify the factors associated with smokers’ learning preferences. A further question is related to the impact of the game on smokers’ later use of other proposed tools and aids for smoking cessation. It may also be helpful to study and develop other varieties of the game, as differences in game format, content, or purpose may change its clinical impact and attractiveness. A further study, in preparation, will assess an electronic version of the game, which may be more appealing in general, especially for younger smokers.

## Competing interests

No competing interests. The first author is the author of the Pick-Klop game.

## Authors’ contributions

Y. Khazaal wrote the game. R. Prezzemolo, G. Monney, and A-S. Protti contributed to improvements in the game. Y. Khazaal and Daniele Zullino designed the study. A. Chatton undertook the statistical analysis. Y. Khazaal, D. Zullino, and A. Chatton contributed to the writing of the manuscript. J Cornuz, J-F Etter, R. Khan, F. Zebouni, and Y. Edel contributed to the editing and review of the final manuscript. The other authors carried out the research. R. Prezzemolo, J. Jacquet, O. Ruggeri, E. Burnens, G. Monney, and A-S Protti carried out the research and contributed to data collection. All authors contributed to and have approved the final manuscript.
